# Sailing through the southern seas of air–sea CO_2_ flux uncertainty

**DOI:** 10.1098/rsta.2022.0064

**Published:** 2023-06-26

**Authors:** Peter Landschützer, Toste Tanhua, Jacqueline Behncke, Lydia Keppler

**Affiliations:** ^1^ Department Research, Flanders Marine Institute (VLIZ), 8400 Ostend, Belgium; ^2^ The Ocean in the Earth System, Max Planck Institute for Meteorology, 20146 Hamburg, Germany; ^3^ GEOMAR Helmholtz Centre for Ocean Research Kiel, 24148 Kiel, Germany; ^4^ International Max Planck Research School on Earth System Modelling, 20146 Hamburg, Germany; ^5^ Scripps Institution of Oceanography, University of California San Diego, La Jolla, CA 92093, USA

**Keywords:** Southern Ocean, observations, carbon dioxide

## Abstract

The Southern Ocean is among the largest contemporary sinks of atmospheric carbon dioxide on our planet; however, remoteness, harsh weather and other circumstances have led to an undersampling of the ocean basin, compared with its northern hemispheric counterparts. While novel data interpolation methods can in part compensate for such data sparsity, recent studies raised awareness that we have hit a wall of unavoidable uncertainties in air–sea ${\mathrm{CO}}_{2}$ flux reconstructions. Here, we present results from autonomous observing campaigns using a novel platform to observe remote ocean regions: sailboats. Sailboats are at present a free of charge environmentally friendly platform that recurrently pass remote ocean regions during round-the-globe racing events. During the past 5 years, we collected >350 000 measurements of the sea surface partial pressure of ${\mathrm{CO}}_{2}$ (p${\mathrm{CO}}_{2}$) around the globe including the Southern Ocean throughout an Antarctic circumnavigation during the Vendée Globe racing event. Our analysis demonstrates that the sailboat tracks pass regions where large uncertainty in the air–sea ${\mathrm{CO}}_{2}$ flux reconstruction prevails, with regional oversaturation or undersaturation of the sea surface p${\mathrm{CO}}_{2}$. Sailboat races provide an independent cross-calibration platform for autonomous measurement devices, such as Argo floats, ultimately strengthening the entire Southern Ocean observing system.

This article is part of a discussion meeting issue ‘Heat and carbon uptake in the Southern Ocean: the state of the art and future priorities’.

## 1. Introduction

The Southern Ocean is the dominant marine sink for anthropogenic carbon emitted since the beginning of industrialization [1,2]. Being responsible for roughly 40–50% of the annual oceanic ${\mathrm{CO}}_{2}$ uptake [3] due to its size, the Southern Ocean, south of 35${\;}^{\circ }$S plays a crucial role in mitigating climate change. Despite being responsible for the largest proportion of the global oceanic ${\mathrm{CO}}_{2}$ uptake, the Southern Ocean remains the most controversial ocean basin with regard to ${\mathrm{CO}}_{2}$ fluxes and its regional uptake and release of ${\mathrm{CO}}_{2}$ from/to the atmosphere. Various methods, ranging from ocean biogeochemical models through observation-driven mapping methods that estimate the oceanic ${\mathrm{CO}}_{2}$ sink [4,5], yield large differences in the magnitude of the Southern Ocean carbon uptake, as well as its variability [4,6,7].

**Figure 1.**
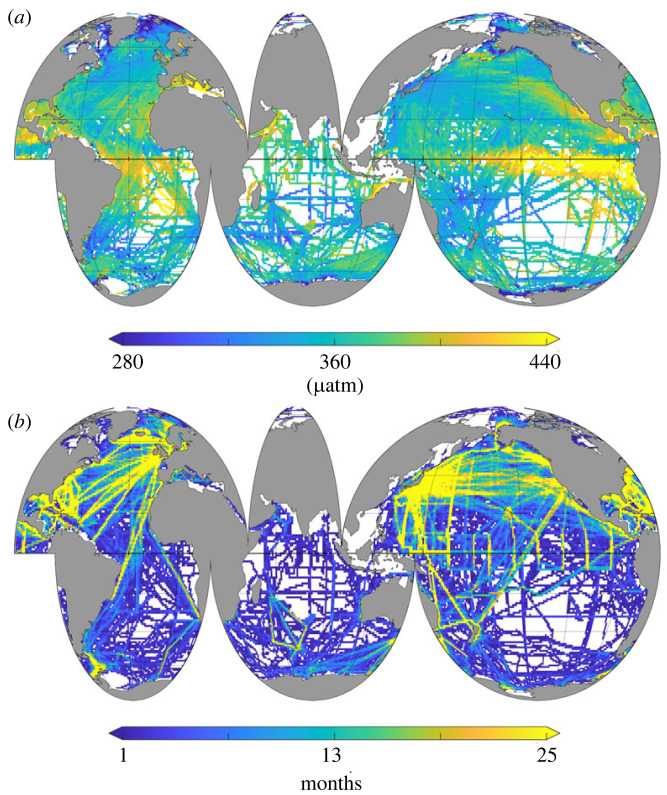
The data distribution from the Surface Ocean ${\mathrm{CO}}_{2}$ Atlas database. (a) The long-term mean f${\mathrm{CO}}_{2}$ map from all measurements derived from the gridded product within the SOCAT database v2022. (b) The number of months with at least one observation within each 1×1 degree pixel since 1957.

Studies suggest that these discrepancies are largely the result of the data sparsity in the Southern Ocean [8,9]. Our current observing system of the global sea surface partial pressure of ${\mathrm{CO}}_{2}$ (p${\mathrm{CO}}_{2}$), from which the air–sea ${\mathrm{CO}}_{2}$ flux can be inferred using a bulk gas transfer calculation [10,11], is heavily biased towards the Northern Hemisphere [12]. This is to a large degree the result of the implementation of the Voluntary Observing Ship (VOS) programme and the subsequent increase of repeat ${\mathrm{CO}}_{2}$ measurements along common shipping routes in the north. In the Southern Hemisphere, by contrast, few shipping lines that take ${\mathrm{CO}}_{2}$ measurements exist, resulting in many sparsely or even entirely unobserved ocean areas in the Southern Ocean, which are visible in the Surface Ocean ${\mathrm{CO}}_{2}$ Atlas (SOCAT) database, i.e. the largest collection of underway p${\mathrm{CO}}_{2}$ and sea surface fugacity of ${\mathrm{CO}}_{2}$ (${\mathrm{fCO}}_{2}$) measurements [12] (figure 1).

In the recent past, emerging methods based on machine learning were used to overcome data limitations and to fill gaps in the ${\mathrm{CO}}_{2}$ measurement network [13,–17]. The advantage of machine learning methods over commonly used statistical interpolations is that they ‘learn’ from the full set of measurements and auxiliary driver data, i.e. they draw information from the well-observed Northern Hemisphere or elsewhere to fill data gaps in sparsely sampled areas without being dependent on autocorrelation length scales between measurements (usually of the order of 400 km globally [18]). This has led to a significant increase in global, mapped observation-based air–sea ${\mathrm{CO}}_{2}$ flux estimates which have also made their way in the annual Global Carbon Budget [5] and recent IPCC reports [19].

On closer inspection, [8] found that while testing several newly emerging machine learning techniques, the bias and root mean squared deviation towards independent measurements is not improving, regardless of the method used. Instead, the authors hypothesized that we have reached a threshold, and the authors refer to this as ‘the wall’. Irrespective of the method used, we are currently limited by the available information, particularly in the Southern Ocean, and the only way to go beyond this wall is to increase the number of observations. This view is supported by [9], who show that data sparsity is the main source of uncertainty in the air–sea ${\mathrm{CO}}_{2}$ flux of the Southern Ocean, particularly when decadal variations are considered. These uncertainties can be substantially reduced by increasing the number of measurements [9,20,21], illustrating that without building extra observational capacities, carbon budgeting exercises might be in jeopardy.

To overcome the data limitation in the Southern Ocean, the Southern Ocean Carbon and Climate Observations and Modelling project (SOCCOM) has set out to improve the number of carbonate system observations [22].

By using autonomously sampling robotic floats (Argo floats), we can directly observe pH and salinity, which are subsequently used to estimate the sea surface p${\mathrm{CO}}_{2}$ usually using a locally interpolated alkalinity regression (LIAR) algorithm [23,–25] and ${\mathrm{CO}}_{2}$ system calculations [26].

The first results from the floats were eye-openers: in austral winter, the float-based estimates of the air–sea ${\mathrm{CO}}_{2}$ flux detected a stronger outgassing signal than previous estimates, putting the magnitude of the ${\mathrm{CO}}_{2}$ flux inferred from ship-based observations in question [24], noting that the majority of Southern Ocean ship-based observations are from the summer months. Combining ships and floats, [26] similarly revealed a strong wintertime outgassing signal, however, weaker than when only floats are considered in the analysis. One remaining caveat, however, exists: because the floats do not measure the sea surface ${\mathrm{pCO}}_{2}$ directly, an inevitable measurement uncertainty of about 11 μatm is introduced [23,27], and it is yet to be clarified, how much of the discrepancy between ship-based and float-based air–sea ${\mathrm{CO}}_{2}$ fluxes in the Southern Ocean is explained by this uncertainty. Furthermore, float-based observations have a different spatial and temporal footprint compared with ships, i.e. one point in time and space every 10 days for floats, which complicates the assessment of the difference.

Since the start of SOCCOM, a few other novel and promising ways to increase highly accurate ${\mathrm{pCO}}_{2}$ measurements have been developed. In 2019, Saildrones, which are autonomous floating platforms, equipped with high-accuracy ${\mathrm{pCO}}_{2}$ instrumentation, have completed their first Antarctic circumnavigation [28]. Working with autonomous vehicles like Saildrones has a large potential, in particular for winter-time missions, but these are—although an order of magnitude more cost efficient than research vessels—associated with daily costs in excess of $2500, and unlike the hydrographic surveys, no long-term deployment planning currently exists. Other platforms, like moorings, drifters and so on, have equally been deployed to fill the Southern Ocean data void; however, the lack of regular calibration remains their main shortcoming.

One additional fleet that has thus far received little attention despite forming the majority of ships in the global ocean is sailboats. Unlike cargo ships, sailboats provide the opportunity to collect data from remote oceans at a much lower cost than research ships or Saildrones without compromising on data quality.

During round-the-globe racing events, sailboats reach the most remote ocean regions, regularly circumnavigating the Southern Ocean and other sparsely sampled ocean regions. Currently, major racing events take place on an annual basis, providing the opportunity to survey remote oceans such as the Southern Ocean. In addition, skippers and their teams are increasingly encouraged to support science, and racing event organizers, such as The Ocean Race or the Vendée Globe race, are increasing their capacities to support science projects along main racing events.

Here, we present a new ${\mathrm{pCO}}_{2}$ dataset collected over the past 5 years aboard two sailboats globally, and we will highlight a major subset of these data, collected in the Southern Ocean during the Vendée Globe race from November 2020 through January 2021 aboard the race yacht Seaexplorer. The data—all quality controlled and submitted to the Surface Ocean ${\mathrm{CO}}_{2}$ Atlas (SOCAT)—present a rare set of data collected during the circumnavigation of Antarctica in austral summer. We will discuss current knowledge gaps in the Southern Ocean ${\mathrm{CO}}_{2}$ flux estimates regionally and how this and future races have the potential to close these gaps.

## 2. Methods

In 2017, the Sailing meets Science accord was launched, creating a scientific and citizen science partnership with skipper Boris Herrmann to measure carbon dioxide in seawater on his IMOCA 60 class sailboat ‘Seaexplorer - Yacht Club de Monaco’ (former Malizia, i.e. correspondent to the team name) during off-shore racing events. With Fabrice Amedeo and his IMOCA 60 sailboat ‘Newrest - Art & Fenêtres’ (now ‘Nexans - Art & Fenêtres’) in 2018, a second skipper joined the campaign to measure p${\mathrm{CO}}_{2}$ in the open ocean.

**Figure 2.**
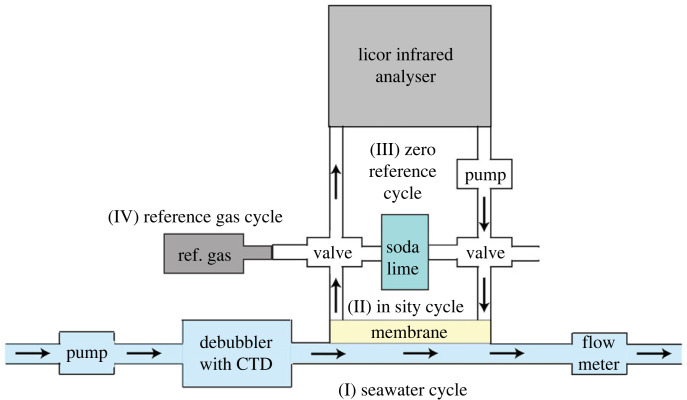
Simplified schematic of the measurement principle of the OceanPack^TM^ Race system.

One speciality of IMOCA 60 boats that offer the opportunity to measure underway in situ ${\mathrm{pCO}}_{2}$ in seawater is the water inlet via the keel, positioned at a depth of 2 m below the sea surface. Seawater enters through the keel and can then be used by an equilibrator system. As size, weight and energy consumption play a significant role for racing sailboats, a particularly lightweight, autonomous and energy efficient but equally sturdy system needs to be in place. For our purpose, we used the OceanPack^TM^ Race system, developed by SubCtech industries (figure 2). A predecessor of this system has been previously used and evaluated aboard research vessels [29,30] and has been previously successfully used on racing sailboats (Volvo Ocean 60’ boats, without foils) deployed during the Volvo ocean race in 2017–2018 aboard ‘Turn the Tide on Plastic’ and ‘AkzoNobel’ in the Southern Ocean. Additional sailboats, not part of the initial Sailing meets Science accord and thus not further discussed, are equipped with the same system.

The measurement principle is illustrated in figure 2. Seawater, entering through the keel (or a thru-hull fitting), at a controlled flow rate of $>5\;{l\;\min}^{-1}$, enters a debubbler, i.e. a cylindrical unit with headspace that prevents bubbles to be further transported along the water cycle. The debubbler unit further includes a conductivity–temperature–depth (CTD) sonde. Since sailboats reach velocities well above 15 knots during races, flying over the oceans on their foils, the debubbler unit prevents air bubbles from reaching the membrane and potentially distorting the ${\mathrm{CO}}_{2}$ measurements. The CTD sonde, located in the debubbler measures seawater temperature and salinity for the final calculations of the sea surface ${\mathrm{pCO}}_{2}$.

At the end of the seawater cycle, a membrane separates the seawater from a closed air loop flowing at a rate of about $500\;{\mathrm{ml}\;\min}^{-1}$. Through the membrane, gases exchange between the air and water sided loops until an equilibrium is reached, while water cannot enter the air-loop through the membrane. Unlike commonly used head-space equilibration systems [31,32], equilibration of the air loop through the membrane is slower [29,30], and hence, it is favourable that the initial disequilibrium is within the range of environmental conditions (i.e. roughly within 100--200 μatm) for a response time within 30 min, based on the obtained field data.

A membrane pump then cycles the air through a LI-COR L840 infrared gas analyser where simultaneously the barometric pressure within the air loop is recorded. The use of a membrane pump is somewhat problematic, as it creates overpressure at the membrane site of the air loop and underpressure at the detector side. To account for this difference, a differential pressure sensor is installed, which records the pressure difference between the detector and the membrane (where the equilibration takes place) for later correction of about 10 hPa in the mean for the measurement cycle.

The most important feature of the measurement system is the underway calibration. Racing events, such as the Vendée Globe race, go on for multiple weeks and months without the opportunity to service the measurement system in between. Likewise, skippers competing in professional racing events do not have the time and capacity to perform maintenance steps aboard. On top of that, the size and weight of the system are a crucial consideration for the sailors not to jeopardize their chances to win a race. Therefore, a two-point calibration is performed once a day. This is following the minimal guidelines of the SOCAT QC cookbook [33] for obtaining an estimated data accuracy within 5 μatm (SOCAT quality flags C,D). Firstly, a zero reference calibration is performed where ${\mathrm{CO}}_{2}$ is stripped out of the air via a soda lime cartridge (figure 2). Secondly, a known reference gas is used to flush the air loop. Currently, the measurement system uses a 2 l bottle where a pre-calibrated air mixture is compressed at up to 300 bar to allow daily calibration over a long period of time without changing the bottle. As the sailboats measure the sea surface ${\mathrm{CO}}_{2}$ content in many different environments (coastal ocean, shelf seas, open ocean and so on), the reference gas bottle is at a value mostly exceeding current atmospheric molar fraction of CO_2_ (x${\mathrm{CO}}_{2}$) values (gas bottle concentrations varying between 400 and 600 ppm over the past years), so the two-point calibration (zero ${\mathrm{CO}}_{2}$ and reference gas) spans the entire range of observed ${\mathrm{CO}}_{2}$ during a racing event.

Numerical data of the sea surface molar fraction of ${\mathrm{CO}}_{2}$ in the air loop (${\mathrm{xCO}}_{2}$), the temperature and salinity from the debubbler, the pressure from the air-loop and other auxiliary parameters (such as flow rate) that are relevant for the data quality control are stored via a data-logger unit at a frequency of 10 s. These data are usually downloaded (via a standard USB port) by the sailors and their teams at sea and transferred via satellite to GEOMAR or the Max Planck Institute for Meteorology, where the quality control takes place and the metadata sheets are prepared alongside the data. The quality control includes a thorough check of all sensor readings and—if possible and available—cross comparison with other ${\mathrm{CO}}_{2}$ measurements, e.g. from other sailboats at the same race. In addition, flow rates are checked and the gas standard and zero readings are investigated for potential drifts. Finally, sailors also report to us things they observe at sea, e.g. blooms, so we can check the ${\mathrm{CO}}_{2}$ readings accordingly and potentially spot problems related to biofouling that could cause the instrument readings to drift [29].

To obtain the partial pressure of ${\mathrm{CO}}_{2}$ at the equilibrator, we multiply the ${\mathrm{xCO}}_{2}$ with the barometric pressure at the LI-COR sensor, corrected to the membrane via the differential pressure sensor. Using the calculations provided in the standard operational procedures guideline by [34], with the temperature and salinity measurements from the CTD unit, we obtain the final value for the fugacity of ${\mathrm{CO}}_{2}$ (${\mathrm{fCO}}_{2}$). As the air loop is neither dried nor vented, we do not perform any moisture correction. In addition, since the length of the water loop is only in the order of 2 m with no ambient warming occurring within the hull of the sailboat, we do assume that the temperature and salinity recorded by the CTD unit reflect the sea surface temperature and salinity without additional temperature correction (e.g. following [35]) and that p${\mathrm{CO}}_{2}$/f${\mathrm{CO}}_{2}$ at the membrane is equivalent to ${\mathrm{pCO}}_{2}/{\mathrm{fCO}}_{2}$ at the sea surface. Before data archival, all measurements are further averaged to 1 min timesteps.

Upon inclusion in SOCAT, the data become part of a 1×1 degree gridded product [36], which is then used for the estimation of the global air–sea ${\mathrm{CO}}_{2}$ flux and its variability in time. Therefore, in the following section and for all visualizations, we equally refer to the measurements on a 1×1 degree grid. In addition, as we do not measure atmospheric ${\mathrm{CO}}_{2}$ on sailboats, we calculate the air–sea p${\mathrm{CO}}_{2}$ difference using the NOAA marine boundary layer (MBL) [37] ${\mathrm{xCO}}_{2}$ product, from which we derive atmospheric pCO_2_ fields (following the calculations outlined in [14]) and from which we calculate the air–sea p${\mathrm{CO}}_{2}$ difference and the air–sea ${\mathrm{CO}}_{2}$ exchange.

## 3. Results

**Figure 3.**
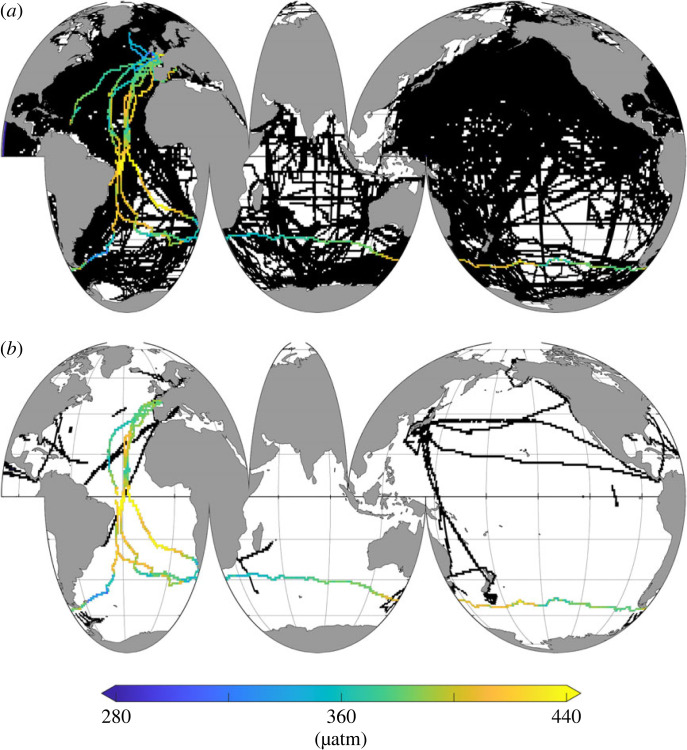
Ship tracks in SOCATv2022 (black background) and from sailboats (colour indicating the measured ${\mathrm{fCO}}_{2}$). (a) All data from 1957 onward and (b) the data from November 2020 through January 2021.

Over the past 5 years, ‘Seaexplorer - Yacht Club de Monaco’ and ‘Newrest - Art & Fenêtres’ have collected >350 000 measurements of sea surface ${\mathrm{pCO}}_{2}$. All these data have been processed as described earlier and have been submitted together with calculated ${\mathrm{fCO}}_{2}$ to the SOCAT database, where they received a flag C, i.e. an expected measurement accuracy within 5 μatm based on the operational procedures. This accuracy is based on the metadata provided and the regular calibration with at least one non-zero gas standard. Although tested with different systems (OceanPack^TM^ CUBE system instead of RACE), field intercomparisons [29,30] support this and suggest that differences with more accurate systems are largely within 5--6 μatm, i.e. within the combined measurement uncertainty. Nevertheless, larger differences at sea have been equally observed [29] that are not fully resolved yet. In total, sailboats contributed to around 0.1% of all measurements collected around the globe within SOCAT; however, with 89 896 measurements obtained in 2020 alone, Seaexplorer was one of the major data contributors to SOCAT and consequently the Global Carbon Budget v2021 [5], particularly in the Southern Ocean (figure 3). Many well-known ocean racing events (Route du Rhum, Transat Jacques Vabre, the Vendée Arctic), shown in figure 3, have contributed to the ${\mathrm{fCO}}_{2}$ data collection. Although since 2017 each and every race has only been included once, these events are recurring, and the map in figure 3 can be redrawn by repeat occupations.

The scientific highlight thus far was the Vendée Globe race, where both Newrest and Seaexplorer participated. The latest Vendée Globe race took place from November 2020 through January 2021 and is a 4-yearly recurring racing event, where IMOCA class 60 boats sail south from the French town of Les Sables d’Olonne in the region Vendée, circumnavigate the Antarctic continent and head back north after passing Drake Passage back to Les Sables d’Olonne. Overall, the time in which sailboats complete this journey varies by days; however, the fastest time recorded in 2020/2021 was just over 80 days. What makes this racing event special—besides the geographical aspect—is that it is a single-hand racing event, which means that only a single skipper is aboard for several weeks and months at sea.

Newrest’s journey around the globe ended unplanned in the South Atlantic but nevertheless collected valuable data in the South Atlantic with a stop in Cape Town for repairs and a return leg to France. Seaexplorer finished the race in fifth place in 80 days 14 hours 59 min and 45 s, during which time the vessel continuously and autonomously collected valuable sea surface ${\mathrm{pCO}}_{2}$ and ${\mathrm{fCO}}_{2}$ data (figure 3). Especially noteworthy is that Seaexplorer sailed through ocean regions where thus far no ${\mathrm{CO}}_{2}$ measurements have been obtained by ships (e.g. in the South Atlantic Ocean and the Indian and Pacific sectors of the Southern Ocean), illustrating the potential to fill remaining measurement gaps with this platform (figure 3).

**Figure 4.**
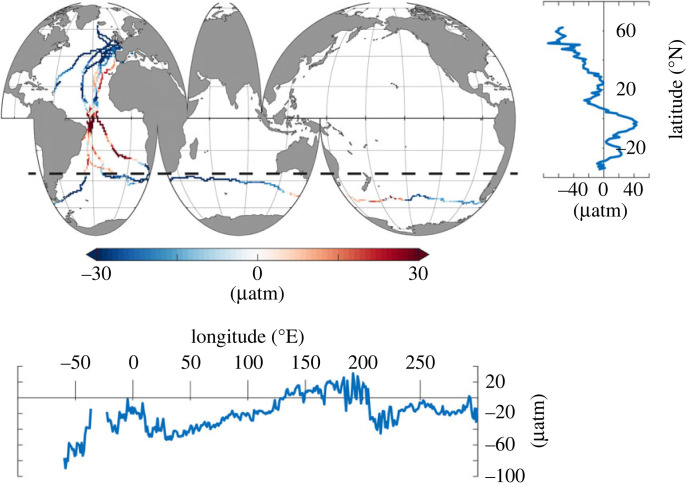
Sea surface delta ${\mathrm{pCO}}_{2}$ (${\mathrm{pCO}}_{2}$ in the surface ocean minus ${\mathrm{pCO}}_{2}$ in the atmosphere) and their zonal and horizontal averages. The zonal mean is reported only north of $35^{\circ }S$ (indicated by the black dashed line), whereas the meridional mean is calculated for the area south of $35^{\circ }$ S.

The results in figure 3 demonstrate that the highest sea surface ${\mathrm{pCO}}_{2}$ is measured in coastal regions, the Canary upwelling system as well as the tropical Atlantic, where the ocean comprises a source of carbon dioxide to the atmosphere, in line with the findings by [38,39]. The lowest p${\mathrm{CO}}_{2}$ and consequently the largest ${\mathrm{CO}}_{2}$ undersaturation and potentially strongest sink were observed during the Vendée Arctic race and the Bermudes 1000 event, both in the North Atlantic Ocean in boreal summer (figure 4), in agreement with the past research [40,41].

In the austral summer of 2020/2021, the Southern Ocean was largely undersaturated with respect to atmospheric ${\mathrm{CO}}_{2}$ along the measurement track (figure 4). The only exception can be observed polewards of Tasmania and New Zealand, where the ocean is supersaturated. In addition, significant amounts of ${\mathrm{pCO}}_{2}$ variability can also be observed occurring along the sail track in the Southern Ocean.

The comparison earlier illustrates the potential to substantially increase the amount of available measurements in currently underrepresented ocean regions and the possibility to study regional features. The question posed here though is whether continuous reoccupation of global racing events and continuation of ${\mathrm{CO}}_{2}$ measurements have the potential to break, or sail through, the current ‘wall’ of uncertainties we are facing [8]. To this extent, we use a two-step neural network approach (SOM-FFN) described in [14] that maps the sea surface ${\mathrm{pCO}}_{2}$ and subsequently estimates the air–sea ${\mathrm{CO}}_{2}$ exchange using a bulk flux transfer formulation. We then compare two interpolations in particular, one, where the sea surface ${\mathrm{pCO}}_{2}$ map includes the new data from sailboats in the Southern Ocean in the training, and a second, where the neural network does not include these Southern Ocean data in the training. These two datasets are equivalent to the data submitted and presented in the Global Carbon Budgets v2020 [7] and v2021 [5] by this method.

Comparing two globally integrated air–sea ${\mathrm{CO}}_{2}$ flux estimates for the year 2019 from two different SOCAT versions, we find only insignificant difference (given the uncertainty in the mean flux of 36% stemming from the extrapolation of sparse data, and the uncertainty in the kinetic gas transfer—see [42]). The mean sink from the SOM-FFN method is 3.07 PgC/yr in 2019 [7], whereas using additional observations released the following year [5], where Southern Ocean data from the Vendée Globe are included, the global mean sink increases to 3.20 PgC/yr. This is also true for other mapping methods participating in the global carbon budget with the average change between the Global Carbon Budget v2020 and v2021 only being 0.1 PgC/yr, i.e. well within the methods uncertainty and the standard deviation between all database estimates (±0.26 PgC/yr [5]).

**Figure 5.**
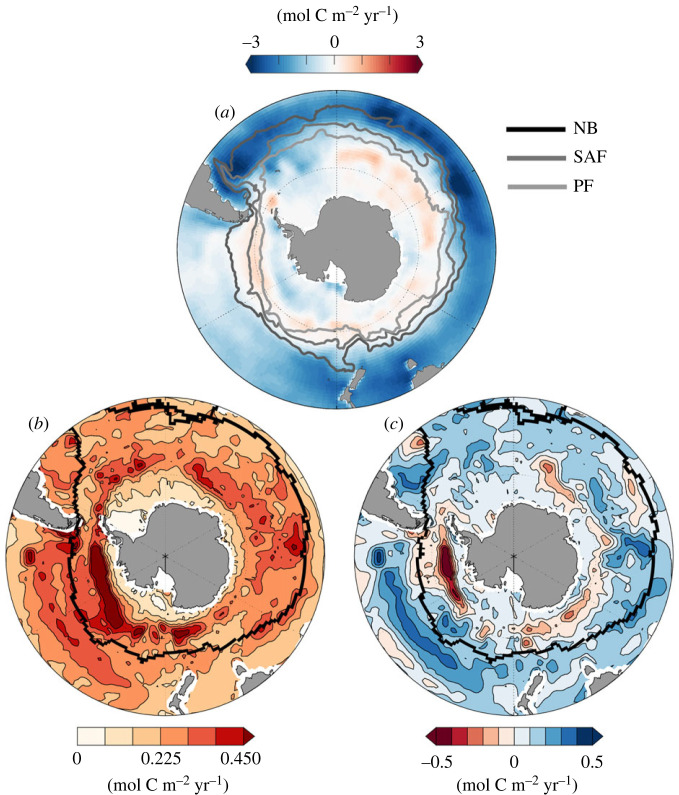
(*a*) Mean air–sea ${\mathrm{CO}}_{2}$ flux from 1982 through 2019. Positive (red) areas show ${\mathrm{CO}}_{2}$ release from the ocean to the atmosphere and vice versa for blue areas. Overlaid on the flux are the major ocean fronts (NB, Northern Boundary; SAF, Subantarctic Front; and PF, Polar Front) from [43]. (*b*) Absolute difference between air–sea ${\mathrm{CO}}_{2}$ flux densities (mean from 1982 to 2019) derived from SOCATv2020 and SOCATv2021 (new sailboat data introduced to SOCAT in the Southern Ocean) and (*c*) as (*b*) but without calculating the absolute difference. Black lines in (*b*) and (*c*) indicate the sailboat tracks during the Vendée Globe race.

Regionally, however, the variance in the air–sea ${\mathrm{CO}}_{2}$ exchange from year to year can be substantial. Figure 5 shows the mean air–sea flux, the absolute difference in the air–sea ${\mathrm{CO}}_{2}$ exchange and the total difference as indication of the direction of change from the SOM-FFN method from 1982 through 2019 between two different versions presented in consecutive Global Carbon Budgets. In addition, major ocean fronts and ship tracks are highlighted in figure 5 as well. Although substantial regionally, negative and positive changes do compensate for each other, leading to only small changes in the integrated net air–sea exchange.

**Figure 6.**
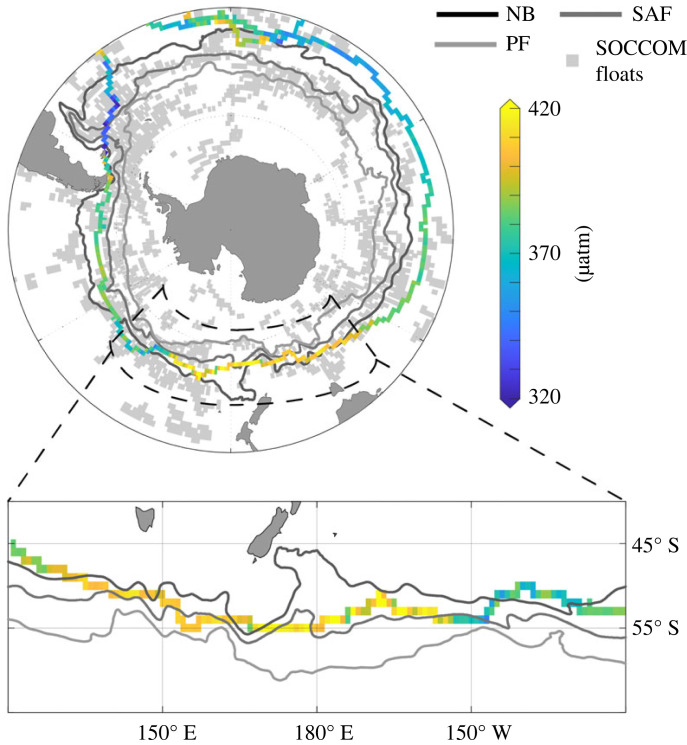
Mean position of the Southern Ocean fronts (NB, Northern boundary; SAF, Subantarctic Front; PF, Polar Front) from [43] and data from Seaexplorer and Newrest as well as SOCCOM float position in the background. The inlay outlined by the black dashed line highlights the area where the highest sea surface p${\mathrm{CO}}_{2}$ was observed during the Vendée Globe race.

Comparing the regions of change with figure 6, it is clear that the majority of the variance observed in figure 5 stems from ACC frontal zones [43] and polewards of the polar front (PF). While sailboats avoid sea-ice, and thus stay north of the Polar Front, we see that Seaexplorer crosses south of the Northern Boundary (NB) and in part the Subantarctic Front (SAF). This is visible zooming into the region south of Tasmania and New Zealand, in figure 6, i.e. the area with a ${\mathrm{pCO}}_{2}$ oversaturation signal and a sharp increase from around 380 μatm to >410 μatm. Zooming into the high p${\mathrm{CO}}_{2}$ region in figure 6, the increase in the ${\mathrm{pCO}}_{2}$ co-locates with the ship entering the interfrontal zones, approaching the subantarctic front. Interestingly, this region is not co-located with the largest change in the air–sea ${\mathrm{CO}}_{2}$ flux (figure 5), likely linked to the fact that it is among the best observed regions in the Southern Ocean [44,45]. However, as the neural network-based mapping informs the global ocean within similar biogeochemical regions, the information south of the Northern Boundary front will inform other, less well-observed regions south of this front.

Recently, many studies have argued for an integrated observing system that consists of many different components [20,21], i.e. ships, Saildrones, Argo floats and moorings to optimally sample the Southern Ocean. Each of these observing platforms has its own strengths and weaknesses, and so a combination of these platforms can draw on the strengths of each. Sailboats have the potential to contribute to such an idealized observing network and support the other observing components. While sailboat races predominantly occur in the respective hemispheric summer season, they will not be able to resolve the present day discrepancy between ship-based and float-based air–sea ${\mathrm{CO}}_{2}$ fluxes in winter [27]; however, the accuracy of the underway system provides the opportunity to cross-calibrate other sensor networks (figure 6). There is a strong overlap between the position of biogeochemical Argo floats from the SOCCOM float array and the track of the Vendée Globe race, particularly north of the Subtropical front in the Pacific and Atlantic sectors of the Southern Ocean. Again, in particular, the Pacific sector on the one hand comprises one of the least well-observed ocean regions from the ship network (figure 1), and on the other hand, this region is among the regions with the largest year-to-year variance in the air–sea ${\mathrm{CO}}_{2}$ flux reconstructions (figure 5). Co-location and cross calibration could therefore strengthen the integrated observing network.

## 4. Discussion

Overall, significant improvements have been made in the past years in quantifying the marine ${\mathrm{CO}}_{2}$ sink [2,13] and in particular the Southern Ocean’s contribution to this sink strength [24,27,28,46,50–]. Less agreement still exists regarding the variability of the ${\mathrm{CO}}_{2}$ sink in the Southern Ocean and its drivers [–27,50,52]. While ongoing measurements from sailboats may not resolve past variations, they may contribute to improve the future monitoring network. One way to resolve discrepancies in past assessments is to find novel ways to use the existing observing network [53]. A rather unexplored field is the adoption of the neural network methods from the surface to the ocean interior, which is currently limited to climatologies [54,55] but can potentially provide a second line of evidence to resolve the Southern Ocean ${\mathrm{CO}}_{2}$ sink strength.

Here, we present a new observing platform that has the potential to fill the data void in remote ocean regions. While free of charge to the scientific community and environmentally friendly, sailboats provide ${\mathrm{pCO}}_{2}$ measurements of reasonable accuracy for Carbon Budget exercises during offshore racing events. While sailboats do not contribute to the closure of the winter data void, they could do so indirectly, by providing a cross-calibration point for robotic sampling methods such as biogeochemical Argo floats and year-round mounted moorings.

Besides the benefits, there are a number of disadvantages of the sailboat measurements that need to be discussed further. Disadvantages of the system include that there are clear assumptions made. The boat design of an IMOCA 60 does not allow for a temperature sensor to be mounted at the seawater inlet; nevertheless, considering the steady flow rate, the short water loop (less than 2 m from the water inlet to the debubbler unit) and the lack of ambient warming, we assume that the lack of inlet temperature has a minimal effect on our final calculations. In addition, the data obtained currently only fulfil the minimal requirements considered by SOCAT for flag C and D, which are, nevertheless, still an improvement towards indirectly calculated ${\mathrm{pCO}}_{2}$ estimates from other carbonate system measurements. Additional reference gases and more frequent calibration (i.e. more than once a day) would therefore be beneficial; however, size and weight restrictions currently prevent us from increasing the number of reference gases or from increasing the reference bottle size.

Another shortcoming is the adjustment time of the membrane. While this can be easily controlled after a reference gas ‘shock’, i.e. a state where the air loop is artificially put into a state of disequilibrium with the seawater, natural ‘shocks’, e.g. crossing steep fronts or mesoscale eddies, result in a slower response time, which needs to be considered when studying short-term and small-scale variations [29,30] compared with faster responding head-space equilibrator systems. A way forward to improve the representation of ${\mathrm{CO}}_{2}$ gradients and to reduce the measurement uncertainty would be to better quantify the adjustment time of the membrane, through laboratory comparisons with other, fast responding instruments.

## 5. Conclusion

Here, we present the first results from 5 years of underway ${\mathrm{pCO}}_{2}$ and ${\mathrm{fCO}}_{2}$ measurements collected on board sailboats during offshore racing events. In total, we were able to collect >350 000 measurements, predominantly in the Atlantic Ocean and the Southern Ocean. These are all made public through the Surface Ocean ${\mathrm{CO}}_{2}$ Atlas (SOCAT) [12]. Through a membrane equilibrator system, regularly calibrated with a zero and a non-zero reference point, the measurement system is durable, lightweight and of sufficient quality to be integrated in air–sea ${\mathrm{CO}}_{2}$ flux studies.

One major advantage of sailboats as platforms is the repeat occupation. With the Vendée Globe race and The Ocean Race both recurring every 4 years, these two major sailing races have already contributed to ${\mathrm{CO}}_{2}$ measurements in the Southern Ocean. We also note that there are other races such as the Clipper Round the World Race, the Jule Verne Trophy and recreational yachts plying Southern Ocean waters that can potentially contribute. Sailboat racing events, and in particular round-the-globe recurring racing events, are good platforms to study temporal changes in remote ocean regions such as the Southern Ocean and to break through the current ‘wall’ of unavoidable uncertainties in air–sea ${\mathrm{CO}}_{2}$ flux reconstructions.

The first 5 years of measurements show that sailboats are not only providing essential data where ships have not measured the sea surface ${\mathrm{CO}}_{2}$ in the past 70 years but they also provide a valuable platform for process studies, as they provide essential high-frequency data from frontal crossings, mesoscale eddies and major blooms, e.g. on the Patagonian shelf. Understanding these processes related to the marine carbon cycle will provide knowledge needed to improve Earth System Models as they push towards increasing resolution. Ultimately, the data from sail boats will help to accurately quantify the oceanic ${\mathrm{CO}}_{2}$ sink and thus provide important information towards reaching climate mitigation and ${\mathrm{CO}}_{2}$ emission reduction goals. In addition, sailboat data can be used to cross-calibrate and compare float measurements. Due to the high-frequency of the sailboat observations, studies focusing on the meso- and submesoscale can also be conducted with these observations, to better understand the processes on these scales.

Finally, sailboats are a low-cost solution where the only costs necessary to derive data from remote ocean regions stem from the measurement system (of the order of €60 k--70 k), in addition to the cost of calibration gases, maintenance and QC efforts. The measurement platform, i.e. sailboats, are not only free of charge to the scientific community, as their prime interest is the competition in racing events, but they are equally an environmentally friendly, emission-free carrier platform. As skippers become increasingly environmentally aware and engaged and as sponsors are equally interested in low emission solutions, future racing events provide the perfect basis to equip fleets of sailboats and significantly increase the ${\mathrm{CO}}_{2}$ measurements in the remote Southern Ocean and other regions.

## Data Availability

All data used and discussed in this article are freely available via www.socat.info and NCEI OCADS (https://www.ncei.noaa.gov/access/ocean-carbon-acidification-data-system-portal/). The interpolated air-sea CO_2_ flux maps from figure 5 can be obtained from: https://www.ncei.noaa.gov/access/ocean-carbon-acidification-data-system/oceans/SPCO2_1982_present_ETH_SOM_FFN.html.
